# El valor de un ajuste oclusal: ¿por qué?, ¿cómo? y ¿cuándo? reporte de caso

**DOI:** 10.21142/2523-2754-1003-2022-122

**Published:** 2022-09-28

**Authors:** Leslie Nicole Garcia-Cahuana, Joselin Harley Martinez-Aparcana, David Juan Figueroa Pastrana, Katherine Joselyn Atoche-Socola

**Affiliations:** 1 Carrera de Estomatología de la Universidad Científica del Sur. Lima, Perú. lesliegarciacah@gmail.com, joselinharley@gmail.com Universidad Científica del Sur Carrera de Estomatología Universidad Científica del Sur Lima Peru lesliegarciacah@gmail.com joselinharley@gmail.com; 2 División de Rehabilitación Oral de la Universidad Científica del Sur. Lima, Perú. dfigueroap@cientifica.edu.pe, kattyas.22@gmail.com Universidad Científica del Sur División de Rehabilitación Oral de la Universidad Científica del Sur Lima Peru dfigueroap@cientifica.edu.pe kattyas.22@gmail.com

**Keywords:** ajuste oclusal, desgaste dental, guías oclusales, occlusal adjustment, occlusal wear, occlusal guidances

## Abstract

El objetivo de la odontología es estabilizar el sistema estomatognático (dientes, periodonto, músculos y articulación temporomandibular), el cual debe trabajar en armonía. Para ello, debe brindar un óptimo cuidado, desde el diagnóstico y la planificación hasta el tratamiento. Sin embargo, en la actualidad, muchos clínicos le dan poca importancia a la identificación de contactos prematuros o interferencias antes de realizar cualquier tratamiento odontológico, lo que debería tomarse en cuenta para brindar una mejora en la estabilidad del cierre mandibular, y la correcta guía anterior y canina en los movimientos mandibulares.

El presente caso clínico describe el tratamiento de un paciente con diagnóstico de desorden funcional oclusal por contacto prematuro, cuyo tratamiento consistió en realizar un ajuste oclusal por desgaste selectivo, siguiendo la técnica descrita por el autor Klineberg, con el objetivo de preservar al máximo la estructura dentaria, mantener el control durante el ajuste oclusal y devolver el mayor número de contactos puntiformes y simétricos.

## Abstract (en)

The aim of optimal dentistry is to stabilise the stomatognathic system (teeth, periodontium, muscles and temporomandibular joint). All of these must work in harmony, as together they provide optimal care from diagnosis through planning to treatment. However, many clinicians currently give little importance to the identification of premature contacts and/or interferences prior to any dental treatment, which should be taken into account to provide improved stability in mandibular closure, correct anterior and canine guidance in mandibular movements.

This clinical case describes the treatment of a patient with a diagnosis of functional occlusal disorder due to premature contact, whose treatment consisted of an occlusal adjustment by selective wear, following the technique described by the author Klineberg, with the aim of preserving the dental structure as much as possible while maintaining control during the occlusal adjustment and returning the greatest number of symmetrical and pointed contacts.

## INTRODUCCIÓN

La oclusión forma parte del sistema masticatorio, el cual es una unidad funcional compuesta por dientes, periodonto, articulación temporo mandibular (ATM) y músculos, entre los cuales debe existir armonía para que el sistema funcione de manera óptima ^1^. Sin embargo, muchos estudios realizados por diversos autores, como Klineberg y Au ^2^, y Solow ^3^, afirman que el procedimiento de ajuste oclusal (AO) modifica ciertas áreas de uno o más dientes para lograr una mejor estabilidad y distribuir de manera adecuada las cargas durante los movimientos excursivos. Por ese motivo, la mayoría de clínicos optan por realizar dicho tratamiento, debido a sus grandes ventajas, que son la reducción de la actividad muscular ^4^, la reducción de las fuerzas a nivel de la ATM y la mejora la condición gingival y periodontal de los pacientes ^5^.

Si bien es cierto que la evolución y los conceptos del AO no han tenido cambios significativos, no existe un protocolo estandarizado para realizar de manera correcta. Para la detección de puntos de contacto, existen dispositivos como el T-scan y materiales como el Arti-Spray, el papel de articular, la cera, la silicona, entre otros, que le permiten al clínico realizar un correcto AO ^6^; pero aún no están estandarizadas y, debido a eso, el clínico no tiene un buen control de este tratamiento, lo cual tiene como posibles consecuencias las interferencias, que a su vez generan una mayor posibilidad de *chipping* (fractura de cerámicas en coronas) y *cracked* (fractura a nivel de amalgama o del propio diente) ^7^^,^^8^.

Actualmente, muchos clínicos omiten o le dan poca importancia a la identificación de contactos prematuros o interferencias antes de realizar cualquier tratamiento odontológico. Según un estudio realizado por Dib *et al*. ^9^, el AO guiado por computadora, utilizando el T-scan, proporciona buenos resultados al reducir la actividad de los músculos maseteros y disminuir el dolor miofacial, lo que genera confort en el paciente. El estudio realizado por Dommisch *et al*. ^10^ menciona que existe un efecto beneficioso del ajuste oclusal sobre el cambio en el nivel de inserción clínica en pacientes con movilidad dental, además de recomendar que se siga investigando el tema. Por último, el estudio realizado por Solow ^3^ afirma que el AO permite la estabilidad en el cierre mandibular y una correcta guía anterior y canina en las excursiones mandibulares.

Debido a esto, el presente estudio tiene como objetivo mostrar el caso clínico de un paciente de 52 años con diagnóstico de desorden funcional oclusal por contacto prematuro, para lo cual se realizó una remodelación dentaria asociada con un ajuste oclusal por desgaste selectivo, utilizando la técnica descrita por Klineberg.

## REPORTE DE CASO

Paciente de sexo masculino de 52 años, sin antecedentes médicos, acudió al Centro Odontológico de la Universidad Científica del Sur, porque presentaba ruido y dolor al masticar. 

### Examen clínico

En el examen clínico extraoral, se observó asimetría facial del lado derecho, una apertura bucal máxima no asistida de 44 mm con deflexión mandibular izquierda. En la auscultación articular, se identificó clic simple del lado derecho e izquierdo. Mediante la palpación, los músculos se hallaron hipertróficos con dolor leve a nivel de la inserción del músculo masetero del lado izquierdo.

**Figura 1:**
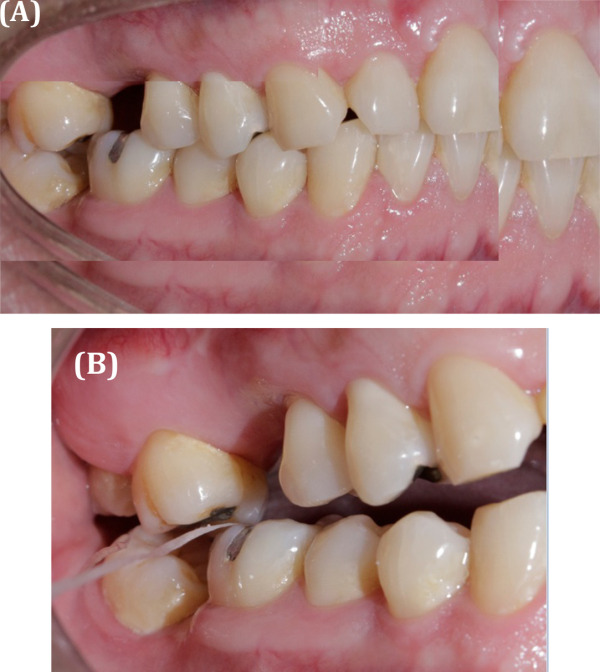
(A) Paciente mordiendo en MIC. (B) Contacto prematuro en RC de la pieza 1.7 cúspide palatina y la pieza 4.8 cúspide distal.

En el examen clínico intraoral, se observó un contacto prematuro entre la cúspide mesiopalatina de la pieza 1.7 con la cúspide disto vestibular de la pieza 4.6, el cual causó un desplazamiento de 1 mm aproximadamente en el plano frontal y de 3 mm en el plano sagital. Asimismo, se observó que dicho contacto causaba interferencias durante los movimientos excursivos. También se identificó atrición y restauraciones en buen estado a nivel cervical, compatibles con posibles abfracciones en múltiples piezas, las cuales eran compatibles con lo indicado en la anamnesis por el paciente, quien afirmó que era apretador dental hace 3 años (Fig. 1). Después de elaborar la historia clínica, los modelos de estudio y los exámenes auxiliares, se obtuvo un diagnóstico definitivo, el cual fue desorden funcional oclusal por contacto prematuro. El plan de tratamiento fue realizar un ajuste oclusal por desgaste selectivo y la confección de una férula oclusal miorrelajante. 

### Elaboración de guías de acetato

**Figura 2:**
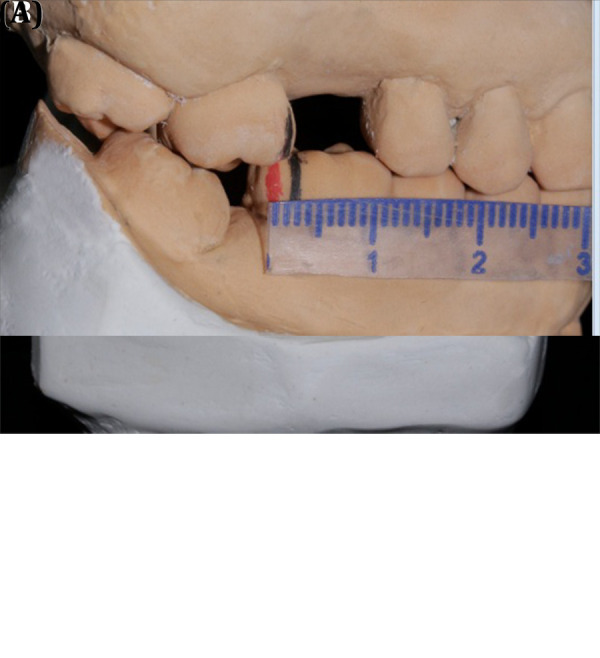
(A) Modelo de estudio articulado en ASA, se observa el deslizamiento sagital de 3 mm. (B) Modelo de estudio con la guía de acetato.

Se tomaron dos juegos de modelos, los cuales fueron vaciados en yeso tipo 4, para lo cual uno fue montado en un articulador semiajustable BioArt, como modelo de diagnóstico. Se realizó un previo ajuste oclusal con un bisturí #15 y papel de articular de 24 micras (µm) (Fig. 2).

El segundo juego de modelos se vació en forma de herradura siguiendo la técnica descrita por Klineberg, para lo cual se confeccionó una lámina de acetato de 0,6 mm en un vacumm. Posteriormente, se marcaron los límites de la guía con un plumón indeleble para su posterior recorte con un disco de carburundum.

**Figura 3:**
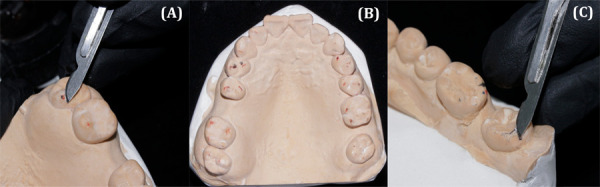
(A) Desgaste con bisturí #15 de la cúspide palatina de la pieza 1.8. (B) Modelo superior después de los desgastes con bisturí. (C) Desgaste con bisturí #15 de la cúspide distolingual de la pieza 4.8.

Después, esta guía se adaptó en los modelos de diagnóstico y se marcaron las zonas donde se había desgastado previamente. Estas zonas marcadas fueron desgastadas usando un fresón multilaminado con forma de flama, y se pulió para evitar molestias en el paciente (Fig. 3).

### Ajuste oclusal por desgaste selectivo

**Figura 4:**
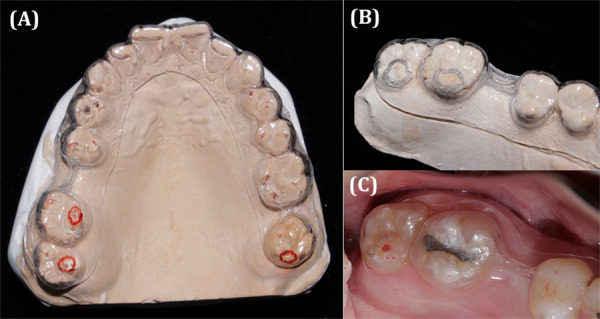
(A) Modelo superior con la guía de acetato marcada en las zonas previamente desgastadas. (B) Modelo superior con la guía de acetato desgastada. (C) Prueba de la guía de acetato en el paciente; se observa sobresalir las zonas que causan interferencia.

En una siguiente cita, se realizó el ajuste oclusal. Primero, se probó en el paciente la guía de acetato, se adaptó y se instaló para, luego, observar cómo las zonas que causaban interferencia sobresalían de la lámina de acetato (Fig. 4). Estas fueron desgastadas siguiendo una secuencia con fresa pimpollo diamantadas FGM de grano fino y extrafino.

**Figura 5:**
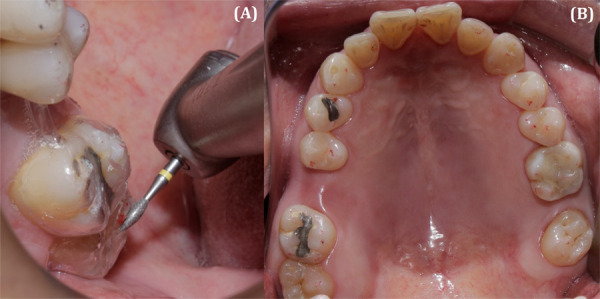
(A) Desgaste de las zonas que causan interferencia con fresa pimpollo fina y extrafina. (B) Prueba de oclusión; se observan puntos de contacto puntiformes, bilaterales y de igual intensidad en todas las piezas dentarias.

Se probó la oclusión con papel articular AccuFilm de 24 um y se observó puntos de contacto en todas las piezas dentarias. Finalmente, se pulió con cauchos de resina y se aplicó flúor barniz para evitar la sensibilidad (Fig. 5).

## DISCUSIÓN

La presencia de un punto de contacto prematuro que genere interferencias en los movimientos excéntricos mandibulares de un paciente podría generar, en el futuro, diversos problemas en el sistema estomatognático, los cuales pueden ocasionar daños a nivel periodontal e, incluso, manifestar signos o síntomas de disfunción temporomandibular ^(10, 11)^. En el presente reporte de caso, se muestra a un paciente diagnosticado con desorden funcional oclusal por contacto prematuro, al que se le realizó un ajuste oclusal por desgaste selectivo, para devolverle un solo arco de cierre y una correcta guía canina, ya que algunas investigaciones han demostrado que este tipo de guías protegen al sistema estomatognático al reducir la actividad y la tensión muscular en posiciones excéntricas ^(^^12^^,^^13^^)^.

Actualmente, la técnica de ajuste oclusal para devolver una correcta guía canina viene siendo utilizada en casos de pre y postratamiento, debido a sus grandes beneficios, como una mejor coordinación y armonía de movimientos musculares, una mayor área periodontal para la recepción de fuerzas y la presencia de contactos oclusales balanceados, ya que ciertas interferencias oclusales podrían ser un factor de alteración en la actividad muscular y articular, lo cual ha sido demostrado desde la década de los 80 ^(^^12^^,^^14^^)^. Asimismo, Solow ^3^ presenta un reporte de caso en el que se realizó una terapia de ajuste oclusal a un paciente que presentaba dolor dental y mordida desigual, lo que demostró que se trata de un tratamiento ideal para controlar las fuerzas negativas que afectan al sistema estomatognático y, a su vez, optimizar los tratamientos posteriores. Esta investigación es reforzada por otros autores ^15^^,^^16^, los cuales mencionan la importancia del diagnóstico de los puntos de contacto prematuro de manera integral en todas las especialidades de odontología para un buen pronóstico de tratamiento.

Se ha demostrado una posible relación entre una fuerza de mordida máxima y la atrición dental como factores causantes de la pérdida de dimensión vertical (DV) ^17^. Existen algunos estudios ^(18, 19)^ que recomiendan realizar un aumento de dimensión vertical en pacientes con atrición dental, según el grado de desgaste y si perdió o no la DV; de esta manera, se mantendría una DV estable y adaptativa. Esta información es reforzada por la investigación de otros clínicos ^17^^,^^20^, quienes mencionan que el aumento de la DV se debe realizar en función de las necesidades del paciente, con algunas consideraciones, como las extrusiones compensatorias de las estructuras dentales remanentes, el espacio disponible para la restauración, la oclusión, la fuerza masticatoria y otros factores como el contorno labial, de sonrisa y la exposición de encía al sonreír. Por tanto, el aumento de la DV no es una alternativa de tratamiento para todos los pacientes.

Es importante realizar el ajuste oclusal en aquellos pacientes que presenten un punto de contacto prematuro, de manera que se genere un equilibrio en el sistema estomatognático con un solo arco de cierre o que se pueda disminuir al mínimo el deslizamiento en céntrica y evitar la función en grupo e interferencias. Así también se evitarán problemas musculares y articulares ^(^^13^^,^^15^^)^.

## CONCLUSIÓN

El diagnóstico y la corrección de los problemas oclusales se trata de manera integral para proporcionar un cuidado óptimo del sistema estomatognático. En el caso de realizar un AO por desgaste selectivo, los modelos de estudio demuestran que resulta de gran utilidad en la etapa de diagnóstica porque ayuda a realizar una buena planificación. En la actualidad, no existe una guía clínica estandarizada que demuestre cuál es la mejor manera de realizar un ajuste oclusal por desgaste selectivo, por eso la técnica descrita por Klineberg nos muestra una forma rápida, sencilla y mínimamente invasiva de realizarlo. Un correcto AO genera estabilidad mandibular, elimina las interferencias, los posibles traumas oclusales y podría disminuir la actividad muscular durante la masticación. Se recomienda una correcta evaluación clínica de los pacientes antes de realizar cualquier tratamiento odontológico, debido a que de esto dependerá la longevidad de los tratamientos y el buen funcionamiento del sistema estomatognático. 

## Financiamiento:

Autofinanciado

## References

[1] Carlsson G, Droukass B (1984). Dental occlusion and the health of the masticatory system. Cranio.

[2] Klineberg I, Au A (2016). Occlusal adjustment in occlusion management.functional occlusion in restorative dentistry and prosthodontics.

[3] Solow RA (2018). Clinical protocol for occlusal adjustment Rationale and application. Cranio.

[4] Aristizábal J, Restrepo de Mejía F, Peralta A, Díaz Y, Triviño A, Ballesteros Y (2017). Bruxism and masseter and temporal muscle activity before and after selective grinding. Int J Odontostomat.

[5] Machado AC, Neto AJF, Junior CDdS, Vilela ALR, Menezes MdS, Teixeira DNR (2018). Influencia do desequilíbrio oclusal na origem de lesao cervical nao cariosa e recessao gengival Análise por elementos finitos. Rev Odontol Bras Central.

[6] Adhuwayhi S (2019). Occlusal indicators a key to achieving stomatognathic system harmony during prosthodontic and restorative treatments - A literature review. Annals of Dental Specialty.

[7] Brandeburski SBN, Vidal ML, Collares K, Zhang Y, Della Bona A (2020). Edge chipping test in dentistry A comprehensive review. Dent Mater.

[8] Li F, Diao Y, Wang J, Hou X, Qiao S, Kong J, Sun Y, Lee ES, Jiang HB (2021). Review of cracked tooth syndrome etiology, diagnosis, management, and prevention. Pain Res Manag.

[9] Dib A, Montero J, Sánchez JM, López-Valverde A (2015). Electromyographic and patient-reported outcomes of a computer-guided occlusal adjustment performed on patients suffering from chronic myofascial pain. Med Oral Patol Oral Cir Bucal.

[10] Dommisch H, Walter C, Difloe-Geisert JC, Gintaute A, Jepsen S, Zitzmann NU (2021). Efficacy of tooth splinting and occlusal adjustment in patients with periodontitis exhibiting masticatory dysfunction: A systematic review. J Clin Periodontol.

[11] Cimic S, Zaja M, Kraljevic S, Simunkovic M, Kopic A, Catic Catic (2016). Influence of occlusal interference on the mandibular condylar position. Acta Stomatol Croat.

[12] Belser UC, Hannam AG (1985). The influence of altered working-side occlusal guidance on masticatory muscles and related jaw movement. J Prosthet Dent.

[13] Miralles R (2016). Canine-guide occlusion and group function occlusion are equally acceptable when restoring the dentition. J Evid Based Dent Pract.

[14] Manns A, Chan C, Miralles R (1987). Influence of group function and canine guidance on electromyographic activity of elevator muscles. J Prosthet Dent.

[15] Pontons-Melo JC, Pizzatto E, Furuse AY, Mondelli J (2012). A conservative approach for restoring anterior guidance a case report. J Esthet Restor Dent.

[16] Afrashtehfar KI, Qadeer S (2016). Computerized occlusal analysis as an alternative occlusal indicator. Cranio.

[17] Jain V, Mathur VP, Abhishek K, Kothari M (2012). Effect of occlusal splint therapy on maximum bite force in individuals with moderate to severe attrition of teeth. J Prosthodont Res.

[18] Nanda A, Jain V, Srivastava A (2011). An electromyographic study to assess the minimal time duration for using the splint to raise the vertical dimension in patients with generalized attrition of teeth. Indian J Dent Res.

[19] Manns A, Valdivieso C, Rojas V, Valdés C, Ramírez V (2018). Comparison of clinical and electromyographic rest vertical dimensions in dolichofacial and brachyfacial young adults A cross-sectional study. J Prosthet Dent.

[20] Abduo J, Lyons K (2012). Clinical considerations for increasing occlusal vertical dimension a review. Aust Dent J.

